# Combination of fingerprints and MCS-based (inSARa) networks for Structure-Activity-Relationship analysis

**DOI:** 10.1186/1758-2946-5-S1-P17

**Published:** 2013-03-22

**Authors:** Sabrina Wollenhaupt, Knut Baumann

**Affiliations:** 1 Institut für Medizinische und Pharmazeutische Chemie, Technische Universität Braunschweig, Beethovenstr. 55, 38106 Braunschweig, Germany

## 

Structure-Activity-Relationship (SAR) analysis of small molecules is a fundamental and challenging task in drug discovery. The knowledge of these relationships between chemical structure and bioactivity is of high value for the medicinal chemist, e.g., in the lead-optimization process or de-novo-design. In order to analyse SARs, the recognition of molecular similarities is a crucial step due to the assumption that molecules with similar properties or features exhibit similar biological activities [1].

Fingerprint-based similarity is an established concept which is also the basis for the Network-like Similarity Graph approach (NSG) [2]. Advantages of fingerprints are that they are fast to compute and well suited for processing large data sets. On the other hand, the interpretation of the results is difficult and the concept appears less intuitive to a medicinal chemist. A promising and more straightforward alternative is the maximum common substructure (MCS). One drawback is the high computational cost which prevents, for instance, the analysis of large HTS data sets.

To tackle these limitations our new method, inSARa (**i**ntuitive **n**etworks for **S**tructure-**A**ctivity-**R**elationships **a**nalysis), combines MCS similarity with the reduced graph (RG) concept [3] for SAR network generation. By using RGs, a higher degree of abstraction is provided as they supply a conceptual representation of pharmacophoric features of a molecule by reducing groups of atoms to single pseudoatoms while retaining the original topology. Moreover, the probability of scaffold hopping and identifying structurally less similar bioisosteric substructures is increased.

Recently, we could show that fingerprints and inSARa provide complementary information for SAR analysis. By combining both approaches, synergetic effects are expected. This will be demonstrated in this contribution.
